# Impact of Glucocorticoid on a Cellular Model of Parkinson’s Disease: Oxidative Stress and Mitochondrial Function

**DOI:** 10.3390/brainsci11081106

**Published:** 2021-08-22

**Authors:** Silvia Claros, Antonio Gil, Mauro Martinelli, Nadia Valverde, Estrella Lara, Federica Boraldi, Jose Pavia, Elisa Martín-Montañez, María Garcia-Fernandez

**Affiliations:** 1 Department of Human Physiology, Faculty of Medicine, Malaga University, Biomedical Research Institute of Malaga, 29010 Malaga, Spain; silviacg@uma.es (S.C.); mauro.o210431@gmail.com (M.M.); elara@uma.es (E.L.); 2 Department of Pharmacology and Paediatrics, Faculty of Medicine, Malaga University, Biomedical Research Institute of Malaga, 29010 Malaga, Spain; antoniogilcebrian@gmail.com (A.G.); nadiavm@uma.es (N.V.); pavia@uma.es (J.P.); 3 Department of Life Sciences, University of Modena e Reggio Emilia, 41125 Modena, Italy; federica.boraldi@unimore.it

**Keywords:** mitochondria, oxidative distress, hormonal stress, Parkinson’s disease

## Abstract

Stress seems to contribute to the neuropathology of Parkinson’s disease (PD), possibly by dysregulation of the hypothalamic–pituitary–adrenal axis. Oxidative distress and mitochondrial dysfunction are key factors involved in the pathophysiology of PD and neuronal glucocorticoid-induced toxicity. Animal PD models have been generated to study the effects of hormonal stress, but no in vitro model has yet been developed. Our aim was to examine the impact of corticosterone (CORT) administration on a dopaminergic neuronal cell model of PD induced by the neurotoxin MPP^+^, as a new combined PD model based on the marker of endocrine response to stress, CORT, and oxidative-mitochondrial damage. We determined the impact of CORT, MPP^+^ and their co-incubation on reactive oxygen species production (O2^−•^), oxidative stress cellular markers (advanced-oxidation protein products and total antioxidant status), mitochondrial function (mitochondrial membrane potential and mitochondrial oxygen consumption rate) and neurodegeneration (Fluoro-Jade staining). Accordingly, the administration of MPP^+^ or CORT individually led to cell damage compared to controls (*p* < 0.05), as determined by several methods, whereas their co-incubation produced strong cell damage (*p* < 0.05). The combined model described here could be appropriate for investigating neuropathological hallmarks and for evaluating potential new therapeutic tools for PD patients suffering mild to moderate emotional stress.

## 1. Introduction

Parkinson’s disease (PD) is a progressive, multifactorial neurodegenerative disease affecting approximately 1–2% of the population over 65 years of age [1,2]. The pathogenesis of this disease is characterised by the involvement of multiple pathways and mechanisms, such as oxidative distress and mitochondrial dysfunction [3], that ultimately produce a loss of dopaminergic neurons from the substantia nigra [4,5]. This loss leads to bradykinesia and other motor disorders which are key for diagnosis, although the disease comes with other non-motor symptoms, such as cognitive impairment, sleep disorders and depression, which increase disability [3]. Chronically repeated episodes of emotional stress, moreover, seem to have an impact on the development of neurodegenerative diseases such as PD [6,7], and it has been suggested that a dysregulation of the hypothalamic–pituitary–adrenal axis (HPA) occurs in PD [8]. This dysregulation may be involved in triggering, exacerbation or progression of the disease.

The extensive production of reactive oxygen species (ROS) in the brain may provide an explanation for the magnitude of the role that these molecules play in PD. The brain consumes about 20% of the O_2_ supply of the body, and a significant portion of this is converted to ROS [9]. Considerable experimental evidence suggests that ROS species, such as superoxide anion radical (O2^−•^), contribute significantly to the loss of dopaminergic neurons in the PD brain [10,11]. The mitochondrial electron transport chain is the major contributor to ROS production [12,13]: accordingly, oxidative-mitochondrial damage plays an important role in the degeneration of these dopaminergic cells [4,5]. In PD and diverse neurodegenerative disorders, oxidative-mitochondrial damage has been proposed as one of the mechanisms implicated. However, the mechanisms behind neurodegeneration involve a complex network of molecular events and phenomena, to which ROS and the oxidative damage are contributors. Inflammatory and auto-inflammatory events are strongly correlated with the pathogenesis of neurodegenerative disorders leading to brain and neuronal injury [14,15,16].

The stress response involves the activation of multiple systems, the HPA axis being crucial [17,18]. Its activation generates a neuroendocrine cascade that results in the elevation of glucocorticoid levels, cortisol in humans and primarily corticosterone in rats and mice, performing important adaptive functions. While initially adaptive, its extended activation contributes to physiological abnormalities and may be involved in the development of disease states [19]. Glucocorticoids receptors are localised to many brain regions, including the striatum and substantia nigra, regions central to PD pathology [20,21]. Thus, excessive stress, leading to chronically raised glucocorticoids levels, is linked to oxidative damage, neurotoxicity and neurodegeneration of dopaminergic neurons [20] and may have detrimental effects on PD neurodegeneration [22]. Oxidative damage, neurodegeneration and cell death have been observed after the administration of corticosterone in neuronal cell cultures from adult rats [23,24].

In mouse PD models, emotional stress and stress-associated glucocorticoid levels are potential risk factors for neurodegeneration [25,26]. Accordingly, elevated glucocorticoid levels in rodent models worsen motor performance and lead to a greater permanent loss of nigral neurons [27] through increased oxidative distress [28,29]. Therefore, these models of PD, in combination with chronic mild stress exposure and corticosterone treatment, are used to investigate and examine the combined effects of emotional stress and dopaminergic degeneration in PD pathology [25,30].

Combined in vitro PD models with corticosterone (CORT) treatment have not, to our knowledge, been developed. The purpose of this study was to examine the impact of corticosterone administration on a dopaminergic neuronal model of PD induced by the neurotoxin 1-methyl-4-phenylpyridinium (MPP^+^) as a new PD model, based on the marker of endocrine response to stress, corticosterone, and oxidative-mitochondrial damage.

## 2. Materials and Methods

### 2.1. Cell Culture

The SN4741 (RRID:CVCL_S466) dopaminergic neuronal cell line derived from mouse substantia nigra [31] was grown in D-MEM high-glucose, supplemented with 10% FBS, 1% penicillin/streptomycin, and l-glutamine 2 mM (Thermo Fisher Scientific, Waltham, MA, USA) to about 70–80% confluence. Cells were seeded in 6-well plates (200,000 each). For mitochondrial O_2_ consumption and immunocytochemistry, plates were pre-coated with 100 μg/mL of poly-d-lysine. SN4741 cells were exposed to MPP^+^ (200 μM), CORT (0.5 μM) or both (200 μM MPP^+^ + 0.5 μM CORT) (Merck/MilliporeSigma, Burlington, MA, USA) for 2.5 or 6 h, in a modified Locke’s solution (NaCl 137 mM, CaCl_2_ 5 mM, KCl 10 mM, glucose 25 mM, Hepes 10 mM, pH: 7.4) supplemented with 1% penicillin/streptomycin and l-glutamine (2 mM). Since the cell line is a mouse-derived cell line, we used the glucocorticoid CORT. These concentrations were chosen on the basis of the viability experiment shown in supplementary Figure S1.

### 2.2. Cell Viability

Viability was determined by quantifying the release of the intracellular enzyme lactate dehydrogenase (LDH, EC 1.1.1.27) [32]. LDH levels were measured in cell-free culture supernatants after 6 h of incubation using a commercial spectrophotometric assay kit (Randox Laboratories, Crumlin, UK) adapted to an ICubio AutoAnalyzer (ICubio Biomedical Technology, Shenzhen, China). The results are expressed as the percentage of LDH released into the medium relative to total LDH (medium and cells lysed using Triton X™-100).

### 2.3. Determination of Mitochondrial Levels of ROS

Mitochondrial ROS production was estimated by measuring O2^−•^ production after 2.5 h of incubation via flow cytometry using MitoSOX™ Red (Thermo Fisher Scientific, USA), according to a previously published procedure [33,34]. Prior to the end of the incubation period, the cells were labelled with 2.5 μM MitoSox in Locke’s solution for 30 min at 37 °C. The cells were then washed and immediately analysed via flow cytometry using the 585/40 nm (FL2) filter in an AccuriTM C6 flow cytometer (BD Biosciences, Franklin Lakes, NJ, USA). Ten thousand events (cells) were recorded and evaluated using FCS Express 5 software (De Novo Software, Pasadena, CA, USA).

### 2.4. Oxidative Stress Cellular Markers

These markers were evaluated by measuring the cellular level of protein oxidation and the fraction of the antioxidant pool available for further anti-ROS activity in cell homogenates. Six hours after treatment, cells were suspended in buffer (HEPES 10 mM, KCl 10 mM, pH 7.4) with a protease inhibitor cocktail (Thermo Fisher Scientific, USA) and phosphatase inhibitors (Merck/MilliporeSigma, USA) and homogenised in the presence of 0.01% digitonin at 4 °C [23]. Protein concentrations were determined using the Bradford protein assay [35]. Advanced-oxidation protein products (AOPP) were evaluated using a micro-assay adapted to an ICubio AutoAnalyzer [34]. Briefly, 18 µL of sample or chloramine-T (ch-T) standard solutions (400–6.25 µmol/L) was placed in each well of the AutoAnalyzer, followed by the addition of 200 µL of reaction mixture, consisting of 81% PBS, 15% CH_3_COOH and 4% 1.16 mM KI. Absorbance was read at 340 nm. AOPP concentration was obtained based on measured ch-T equivalents. Total antioxidant status (TAS), i.e., the total enzymatic and non-enzymatic antioxidant capacity, was evaluated in cells using a commercial TAS kit (Randox Laboratories, UK) adapted to an ICubio AutoAnalyzer that measures the formation of the radical cation ABTS^•+^™ at 600 nm using ABTS™ in the presence of a peroxidase (metmyoglobin) and H_2_O_2_ [23].

### 2.5. Measurement of Mitochondrial Markers

#### 2.5.1. Mitochondrial Membrane Potential

Mitochondrial membrane potential (mΔΨ) was evaluated after 2.5 h of incubation using the lipophilic cationic probe 5,5,6′,6′-tetrachloro-1,1′,3,3′-tetraethyl benzimidazolcarbocyanine iodide (JC-1), according to a previously described procedure [36]. Cells were incubated in 1 μg/mL JC-1 for 20 min at 37 °C, rinsed twice, detached and immediately analysed using FL1 and FL2 filters in an AccuriTM C6 flow cytometer (BD biosciences, Franklin Lakes, NJ, USA). Ten thousand events (cells) were recorded and evaluated using FCS Express 5 software (De Novo Software). To completely deplete the mΔΨ, valinomycin (1 μM), a potassium ionophore, was used as a control. JC-1 is a lipophilic carbocyanine that exists in a monomeric form and accumulates in mitochondria. In the presence of a high mΔΨ, JC-1 reversibly forms aggregates that, after excitation at 488 nm, fluoresce in the orange/red channel (FL2-590 nm). A collapse of the mΔΨ leads to a decrease in the number of JC-1 aggregates and a subsequent increase in monomers that fluoresce in the green channel (FL1-525 nm). This phenomenon is detected as a decrease in orange/red fluorescence and/or an increase in green fluorescence. Thus, the mΔΨ was estimated from the red/green ratios as the FL2/FL1 ratio of JC1 staining.

#### 2.5.2. Mitochondrial Oxygen Consumption Rate

A Seahorse Bioscience XF24 analyser (Agilent Technologies, Santa Clara, CA, USA) was used to measure the mitochondrial O_2_ consumption rate (OCR) [37,38]. Cells were seeded in the specific 24-well plates (20,000 per well). Before each measurement, the cells were washed with PBS, and 590 µL of Agilent Seahorse XF Base Medium (without phenol red and bicarbonate) supplemented with 1 mM pyruvate and 25 mM glucose was added to each well. Measurements were normalised according to protein concentration (Bradford, England). For OCR experiments, we used the commercial “Seahorse XF cell Mito Stress test kit” (Agilent Technologies, USA) following the manufacturer’s instructions; this kit uses oligomycin, carbonyl cyanide-4-(trifluoromethoxy)phenylhydrazone (FCCP) and rotenone/antimycin as mitochondrial toxins. The sequence of mitochondrial toxins added was oligomycin, 1 µM; FCCP, 0.5 µM; rotenone/antimycin A, 0.5/0.5 µM. The inclusion of rotenone/antimycin A served to measure respiration by non-mitochondrial processes, as these compounds eliminate mitochondrial respiration, which was then subtracted from all OCR values obtained.

### 2.6. Immunocytochemistry Procedure

Cells were fixed by adding methanol, previously chilled to −20 °C, and incubating the plate at −20 °C for 20 min. The wells were washed with PBS, and the coverslips were removed and incubated with a tyrosine hydroxylase (TH) primary antibody (1:5000 *v*/*v*; Merck/MilliporeSigma, USA) in PBS/3%, BSA/0.02%, sodium azide at 4 °C overnight and then incubated with a fluorescent secondary antibody Alexafluor™ (Thermo Fisher Scientific, USA) in PBS/BSA for 30 min at room temperature in the dark. The coverslips were mounted with Fluoromount™ (Merck/MilliporeSigma, USA), and images were acquired using a confocal microscope LEICA SP5 II (Wetzlar, Germany), with excitation at 488 nm and emission at 530–568 nm, and processed using the software LAS AF Lite (Leica Microsystems AG, Wetzlar, Germany).

### 2.7. Neurodegeneration

Neurodegeneration of SN4741 cells was measured using the Fluoro-Jade B™ (FJ) dye (Merck/MilliporeSigma, USA) according to a previously published procedure [23,39]. Six hours after treatment, the cells seeded in 12-well plates (50,000 per well) were fixed using 100% methanol, treated with the dye (final concentration of 0.0004% FJ in 0.1% CH_3_COOH) and gently shaken for 30 min in the dark at room temperature. Increased intracellular fluorescence intensity was measured by an FL600 (Bio Tek Instruments, Winooski, VT, USA) bottom-read mode fluorescence microplate reader with 485/20 and 530/25 excitation/emission filters.

### 2.8. Statistical Analysis

Statistical differences were determined using one-way ANOVA. Pairwise comparisons were performed using a post hoc Newman–Keuls multiple comparison test. Statistical significance was set at *p* < 0.05. For data in which the measuring units were arbitrary (AU), the respective values represent the percentage relative to the control value, unless otherwise specified.

## 3. Results

### 3.1. Oxidative Stress Cellular Markers

**Figure 1:**
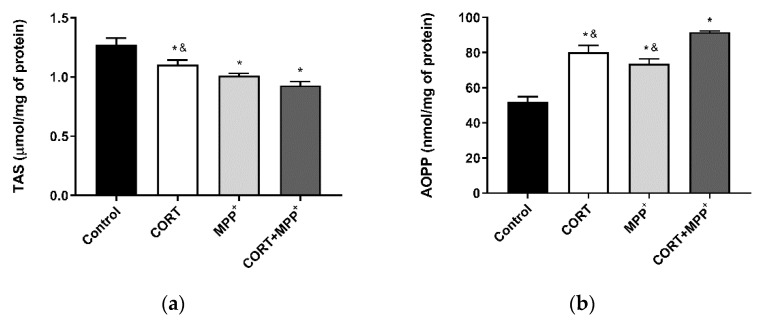
REDOX balance after 6 h of incubation with 0.5 μM corticosterone (CORT), 200 μM MPP^+^ and the combination of the two drugs (CORT + MPP^+^). (**a**) Levels of total antioxidant status (TAS); (**b**) levels of advanced-oxidation protein products (AOPP). Data from three to four independent experiments were combined and presented as mean ± SEM. * *p* < 0.05 compared to control cells; ^&^ *p* < 0.05 compared to CORT + MPP^+^ co-incubated cells.

The TAS, i.e., the fraction of the antioxidant pool available for further anti-ROS activity, was significantly lower in dopaminergic neurons incubated in CORT alone, MPP^+^ alone or their combination (13.3%, 20.6% and 26.9%, respectively) compared to that in control cells (Figure 1a). Incubation with the two drugs also decreased the TAS compared to individual CORT administration (15.7%, *p* < 0.05). We confirmed the expression of the dopaminergic marker TH in the cell cultures, checking that they were composed of dopaminergic neuronal cells (supplementary Figure S2). In order to assess oxidative damage, AOPP were studied in these cells (Figure 1b). AOPP levels were clearly increased in cells treated with CORT, MPP^+^ and their combination (54.4%, 41.83% and 76.58%, respectively) compared to the control cells. Again, the increase in oxidative damage produced by co-incubation with these drugs was significantly higher than in cells incubated with CORT as well as after MPP^+^ administration (12.6% and 19.7%, respectively).

### 3.2. ROS Production and Measurement of Mitochondrial Function

**Figure 2:**
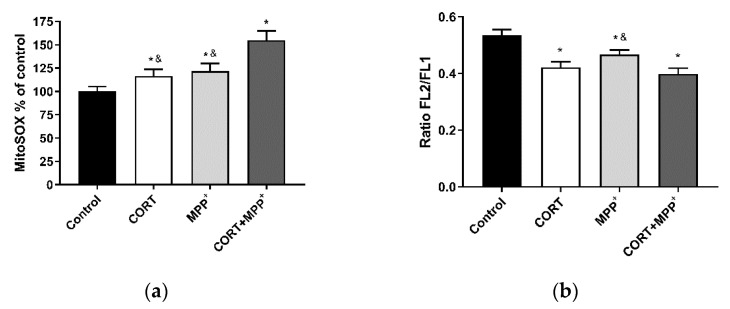
Mitochondrial markers in SN4741 neuronal cells after 2.5 h of incubation with corticosterone (CORT), MPP^+^ and the combination of the two drugs (CORT + MPP^+^). (**a**) Mitochondrial levels of ROS; (**b**) cytofluorometric analysis of mitochondrial membrane potential (mΔΨ). Data were combined from three–four independent experiments and presented as mean ± SEM. * *p* < 0.05 compared to control cells; ^&^ *p* < 0.05 compared with CORT + MPP^+^ co-incubated cells.

Mitochondria are the major source of physiological and pathological cellular ROS. Treatment with CORT alone and MPP^+^ alone produced an increase in MitoSOX™ Red fluorescence (a O2^−•^ indicator) to levels significantly higher than those found in the control cells (16.6% and 21.9% respectively), as shown Figure 2a. Co-incubation with the two drugs dramatically increased free-radical production (54.8%), which was significantly higher than in cells incubated with the drugs individually (24.7% CORT; 21.7% MPP^+^). To assess mitochondrial function, we evaluated mΔΨ and OCR after oxidative damage induced by CORT, MPP^+^ and their combination. Functionally, as can be seen in Figure 2b, incubation of cells with CORT and MPP^+^ individually and the co-incubation with the two drugs, produced significant decreases (21.1%, 12.7% and 25.4%) in mΔΨ versus control cells. It was observed that co-incubation with the two drugs also decreased mΔΨ compared to the administration of MPP^+^ (11.6%, *p* < 0.05).

**Figure 3:**
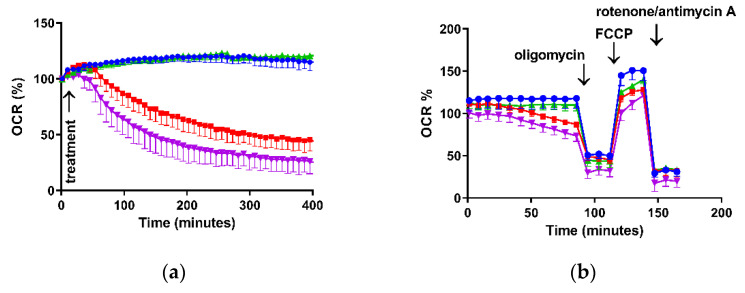
Representative experiments of mitochondrial oxygen consumption rate after incubation with 0.5 μM corticosterone (CORT), 200 μM MPP^+^ and the combination of the two drugs (CORT + MPP^+^) expressed as % of the control. (**a**) OCR time course after treatments; (**b**) measurement of key parameters of mitochondrial function by OCR directly measured using the Mito Stress test kit. Blue line: Control; green line: CORT; red line: MPP^+^; purple line: combination of the two drugs (CORT + MPP^+^).

Regarding O_2_ consumption, Figure 3a shows the OCR time course after incubation of these cells with CORT alone, MPP^+^ alone and the two drugs together. The incubation of the cultures with CORT kept basal respiration at the levels of control cells, whereas the administration of MPP^+^ individually or in combination with CORT significantly decreased OCR after 90 min of incubation (Table 1). These trends continued over the next 5 h.

The bioenergetic assessment is shown in Figure 3b and Table 1; the OCR data obtained in these experiments enabled the determination of O_2_ consumption due ATP synthesis (measured after addition of oligomycin), non-mitochondrial respiration (measured after rotenone/antimycin A administration), O_2_ consumption related to proton leak (residual mitochondrial consumption after oligomycin administration), spare respiratory capacity (SRC—difference between maximal respiration and basal respiration) and maximal respiration (obtained after subsequent addition of oligomycin and FCCP).

Thus, in basal situations, the incubation of cells with MPP^+^ alone and both drugs together showed a significant decrease in OCR of 38.8% and 56.5%, respectively, versus control cells. Regarding O_2_ by mitochondrial ATP synthesis, this decreased by 36.9% (MPP^+^) and 44.4% (co-incubation) compared to the control (*p* < 0.05), whereas we only found differences in the remaining basal respiration not coupled to ATP production (proton leak) after treatment with both drugs. Finally, we observed large decreases in SRC (50–60%) for the three treatment conditions compared to the control cells (*p* < 0.05) as well as in the maximal respiration (26.5% CORT; 43.8% MPP^+^; 50.1% co-incubation, *p* < 0.05). We also observed that the treatments containing MPP^+^ significantly decreased the maximal respiration compared to the individual administration of CORT.

### 3.3. Neurodegeneration

**Figure 4:**
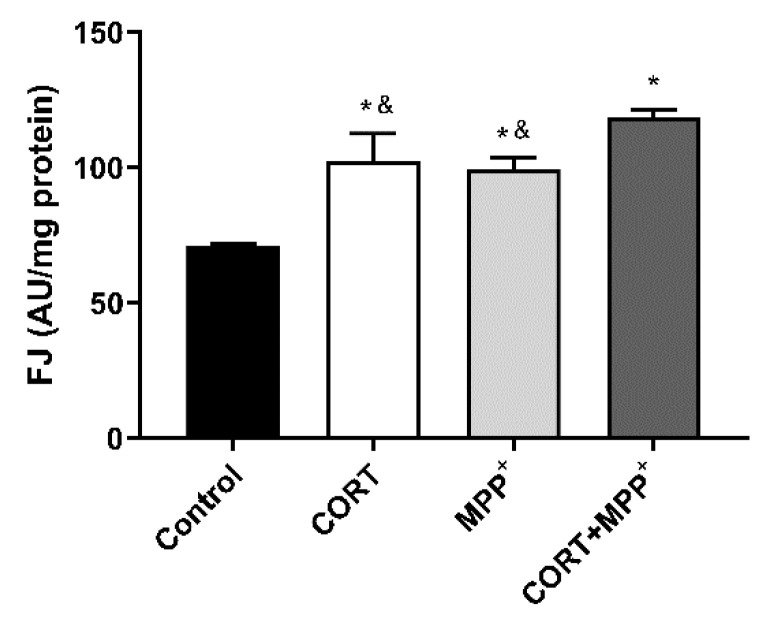
Neurodegeneration after 6 h of incubation with corticosterone (CORT), MPP^+^ and their combination (CORT + MPP^+^). Data were combined from four independent experiments and presented as mean ± SEM. * *p* < 0.05 compared to control cells; ^&^ *p* < 0.05 compared with CORT + MPP^+^ co-incubated cells.

As mitochondrial-oxidative damage plays crucial roles in neurodegeneration, we examined cell degeneration using the polyanionic stain FJ to specifically detect neurodegeneration; this test is specific to a late stage of the degenerative process [39]. Experimentally, our model did not allow us to study prolonged incubation periods due to the poor supplement contained in Locke’s solution. Thus, only short periods (6 h) could be examined, to avoid spontaneous degeneration of the neurons. In these dopaminergic neuronal cells, a significant increase in the FJ intensity of the cells treated with CORT alone, MPP^+^ alone and both drugs was detected compared to the control (44.2%, 40% and 66.8%, respectively), as shown in Figure 4. The increase in FJ intensity produced by the co-administration of these drugs was significantly larger than in cells incubated with CORT or MPP^+^ individually (13.5% and 16%, respectively).

## 4. Discussion

Despite the associations between glucocorticoid levels, mild moderate stress and PD, this relationship in vitro models of glucocorticoid damage and mitochondrial-oxidative damage has not previously been assessed. On the basis of these observations, we decided to research the interplay of CORT and MPP^+^ in dopaminergic neuronal cell cultures as a potential model of PD pathology and mild to moderate emotional stress. We applied a well-established in vitro model of PD induced by the administration of the neurotoxin MPP^+^ [40,41] and combined it with the administration of CORT [24,42].

The results shown in this cellular model of PD pathology based on the marker of the endocrine response to stress, CORT, and the dopaminergic neurotoxin MPP^+^ suggest that the effect of mild to moderate hormonal stress (simulated by CORT administration) aggravates PD pathology (simulated by MPP^+^ administration). Accordingly, the administration of MPP^+^ or CORT individually led to cell damage compared to control situations, whereas the combination of both drugs produced very considerable cell damage, leading to a toxic synergistic effect. As in the case of drug synergism, the potential consequences of concomitant disorders should be re-evaluated, taking their toxic synergistic properties into consideration. The aggravation of PD in conditions of emotional stress has been observed before [43,44], and therefore, the development of tools that help to investigate the relationship between mild to moderate stress and PD and study associated neuropathological hallmarks and potential targets for treatment is crucial.

Our findings point to glucocorticoids as risk factors for PD progression and support previous results obtained either in animal models or in human subjects [26,30,43,44].

In PD and conditions of emotional stress, increases in oxidative distress and mitochondrial damage that may contribute to increased neurodegeneration and/or cell death have been observed [20,22,36,45]. In our model, an increase in free-radical production and cell death was also found, and furthermore co-incubation with the two drugs showed a 2-fold increase in ROS production compared to adding the drugs individually (Figure 2a). This dramatic ROS production may lead to major REDOX imbalance. AOPP may participate in REDOX reactions that could increase cell damage [10,11]. The results in our cellular model indicated a concomitant dramatic increase in AOPP after the combination of the two drug, and a decrease in the TAS levels that partly counteracted the oxidative damage produced (Figure 1). Changes in AOPP and TAS levels agree with our previous results in cellular models of oxidative distress [23,24,34] and with those in cellular models of PD [46].

PD and emotional stress are strongly related to ROS production, with mitochondria being one of the major sources of energy and ROS production [4,5,20,23,24,36]. In this combined model of PD and mild to moderate stress, as in other mitochondrial toxic situations in neuronal cell cultures [23,24,34,38], we found decreases in mitochondrial function measured as mΔΨ and cellular bioenergetic parameters. The current study showed decreases in mΔΨ after the administration of CORT alone and MPP^+^ alone, in agreement with other studies [23,24,40,42], and the effect was potentiated by these drugs’ co-administration (Figure 2b).

Excessive oxidative distress and decrease in mΔΨ alter the energy and the metabolism of neurons leading to dysfunction of mitochondrial biogenesis. Our results show differential effects on cellular bioenergetics after adding the two drugs alone and in combination (Table 1). Whereas basal respiration is decreased after MPP^+^ administration [47], incubation of cells with CORT kept OCR at control levels. However, co-administration of the two drugs enhanced the decrease, showing a provable toxic synergistic effect. The mitochondria-damaging effects of these drugs alone or in combination would deplete energy resources by different mechanisms, contributing to the deterioration of neuronal integrity and function and making neurons less resistant to neurodegeneration [31]. To gain more insight into the mechanisms of how these treatments affect bioenergetics functions, we assessed a number of different aspects [48].

Oligomycin inhibits ATP synthase, and when it is added to the cells, basal OCR decreases. The extent to which it decreases after a treatment can be ascribed to the inhibition in the cell of an ATP-consuming process, of the ATP synthase, or of related proteins or to a decreased ability of the electron transport chain to provide sufficient proton motive force to drive ATP synthesis. In our model, MPP^+^ alone and with CORT inhibited ATP-linked respiration, whereas CORT individually maintained ATP turnover. This effect might represent an adaptive priming to cover the energy supply needed for proper neuronal functioning under conditions of stress [49] leading to a decrease in mitochondrial activity and dysfunction over time. This dual effect of CORT is already known [42]. Concerning the proton leak (remaining basal respiration not coupled to ATP production), co-incubation with both drugs increased this parameter, showing decreased mitochondrial efficiency [47]. In contrast, the maximal respiration decreased in every treatment, dramatically so in MPP^+^ conditions.

The bioenergetic reserve, or SRC, can be used to meet increased energy demands and it is therefore essential to maintain neuronal homeostasis against oxidative and/or other types of cellular stress [50]. In our model, large decreases were observed. The use of mitochondrial SRC by neurons is very variable, ranging from approximately 6–7% in resting situations to up to 80% in firing neurons; thus, deterioration in mitochondrial SRC can be fatal to neurons [50].

Excessive amounts of ROS and mitochondrial dysfunction are among the major mechanisms that trigger neurodegeneration [51]. In fact, we detected large increases in the fluorescence intensity of FJ in dopaminergic neurons incubated with CORT and MPP^+^ individually or after administering a combination of the two drugs (Figure 4). The effect on FJ staining after co-incubation with the two drugs indicated strong neurodegeneration. This result is consistent with those found in animal PD models, where mild stress is considered an environmental risk factor leading to a progressive robust neurodegeneration against a background of PD susceptibility [26]. These models examined the combined effects that emotional stress and relatively progressive dopaminergic neuron loss can have on motor symptoms and nigral cell degeneration. Thus, they revealed that emotional stress caused a worsening of the PD symptoms and accelerated nigral dopaminergic cell degeneration [25,52]. In human studies, this possible relationship between glucocorticoid levels and symptoms in PD has been suggested [8]. There is clear evidence of HPA axis dysfunction and higher cortisol levels in PD, and although studies tend to suggest an association between higher glucocorticoid levels and worsened motor and non-motor symptoms, these findings are mixed [8,53]. This is probably due to the limited number of studies that looked at the links between glucocorticoid levels and clinical disease features of PD, the different samples evaluated in the various articles, and the lack of studies measuring longer-term glucocorticoid release [8]. In this sense, hair cortisone levels may provide additional insights into HPA axis dysfunction in PD [54].

All these findings reveal a central role for mitochondria and the implication of oxidative distress in the relationship between mild to moderate stress and the PD in vitro model proposed, pointing to glucocorticoids as a risk factor for PD progression.

## 5. Conclusions

Stress was long ago hypothesised to contribute to the neuropathology of PD, possibly by increasing the vulnerability of midbrain dopamine cells to degeneration [55]. Given the strong connection between PD and mild to moderate stress, it is imperative to understand their relationship. This in vitro model of PD based on the marker of endocrine response to stress, corticosterone, and the dopaminergic neurotoxin MPP^+^, suggests that the effect of mild to moderate hormonal stress aggravates the PD pathology, with a central role in mitochondrial oxidative stress damage. The in vitro model proposed in this study could be an appropriate model to investigate neuropathological hallmarks and to evaluate potential new therapeutic tools for PD patients suffering mild to moderate emotional stress.

## Figures and Tables

**Table 1:** Study of mitochondrial oxygen consumption.

Conditions	Control	CORT	MPP^+^	MPP^+^ + CORT
Basal respiration	20.9 ± 1.8	21.8 ± 1.1	12.8 ± 1.3 *	9.1 ± 2.1 *
ATP Production	16.0 ± 1.7	17.9 ± 0.9	10.1 ± 1.5 *	8.9 ± 2.1 *
Proton leak	5.4 ± 0.5	4.6 ± 1.1	3.6 ± 0.9	7.4 ± 1.6 *
Spare respiratory capacity	20.2 ± 1.2	8.5 ± 1.4 *	10.4 ± 0.9 *	9.9 ± 3.1*
Maximal respiration	41.1 ± 1.2	30.2 ± 1.8 *	23.1 ± 0.6 *^,&^	20.5 ± 1.9 *^,&^

Data represent the oxygen consumption rate (OCR) in SN4741 neuronal cells assessed in different conditions: basal respiration, after 90 min of incubation with corticosterone (CORT), MPP^+^ and their combination (CORT + MPP^+^). ATP production, measured after addition of oligomycin. Proton leak, considered as the residual O_2_ consumption after oligomycin addition. Spare respiratory capacity, as the difference between maximal respiration and basal respiration. OCR are expressed as pmol/min mg of protein. Data were combined from three independent experiments and presented as mean ± SEM. * *p* < 0.05 compared to control cells; ^&^ *p* < 0.05 compared with CORT incubated cells.

## Data Availability

Data is contained within the article or supplementary material.

## Supplementary Materials

The following are available online at https://www.mdpi.com/article/10.3390/brainsci11081106/s1. Figure S1: Cell death after 6 h of incubation with different concentrations (µM) of corticosterone (CORT), MPP^+^ and their combination (CORT + MPP^+^); Figure S2: Neuronal marker in SN4741 neuronal cells.
