# Supplementary material for: Impact of Glucocorticoid on a Cellular Model of Parkinson’s Disease: Oxidative Stress and Mitochondrial Function
Source: Brain Sci. 2021 Aug 22;11(8):1106. doi: 10.3390/brainsci11081106 (PMC8391158; doi:10.3390/brainsci11081106)
Supplement: Supplementary file 1 [file brainsci-11-01106-s001.zip › brainsci-1337241-supplementary.pdf]

brain sciences

MDPI

Supplementary Material

1. Cell viability

To examine the impact of CORT administration on the dopaminergic neuronal model of PD induced by the neurotoxin MPP^+, we tested different concentrations of CORT, MPP^+ and their co-incubation, for the ability to damage cells without producing notable necrosis or cytotoxicity (Supplementary Figure 1). The doses chosen in the study were 0.5 µM CORT and 200 µM MPP^+, increasing cell death by 14.2% and 19.8% respectively with respect to control. In the case of the co-incubation, a 25.3% increase was found.

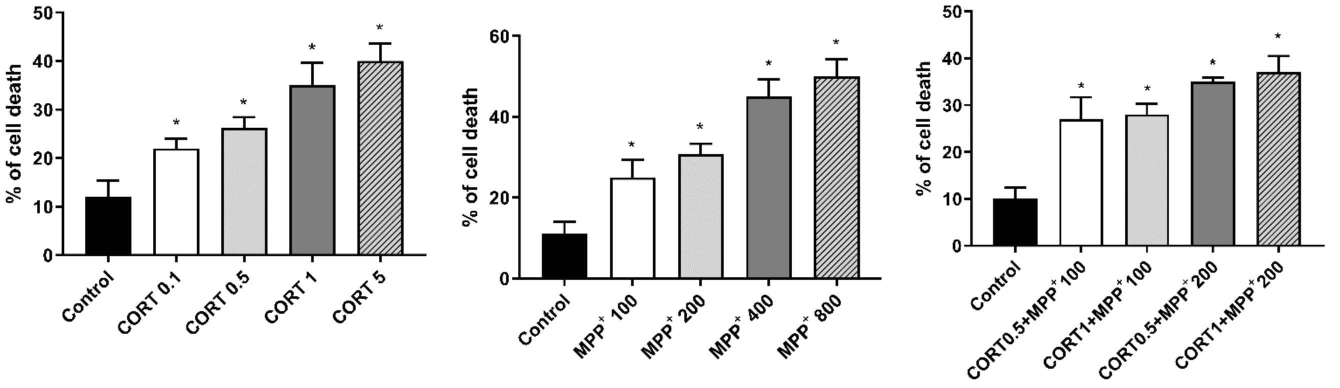

(**a**) (**b**) (**c**)

Figure S1. Cell death after 6 h of incubation with different concentrations (µM) of corticosterone (CORT), MPP^+ and their combination (CORT + MPP^+). (**a**) Effect on cellular viability of different concentrations of corticosterone (CORT); (**b**) Effect on cellular viability of different concentrations of MPP^+; (**c**) Effect on cellular viability of different concentrations of CORT + MPP^+. Data were combined from three to five independent experiments and presented as mean ± SEM. *$p < 0.05$ compared to control cells.

2. Immunocytochemistry procedure

We confirmed the expression of the dopaminergic marker TH in the cell cultures, checking that they were composed of dopaminergic neuronal cells.

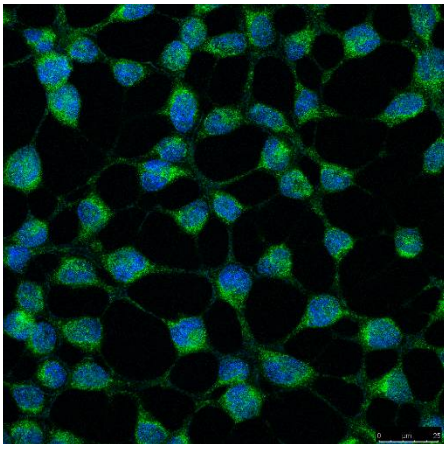

Figure S2. Neuronal marker in SN4741 neuronal cells. Representative immunocytochemistry image of the dopaminergic marker TH in cells.
